# Knowledge, Attitudes, and Practices of Military Personnel Regarding Heat-Related Illness

**DOI:** 10.7759/cureus.49821

**Published:** 2023-12-02

**Authors:** Razan A AlJohani, Naif T Marzook

**Affiliations:** 1 Emergency Department, King Fahad Armed Forces Hospital, Jeddah, SAU

**Keywords:** practices, saudi arabia, military personnel, attitudes, knowledge, heat-related illness

## Abstract

Introduction: Heat-related illnesses are a global concern, affecting millions of people and leading to numerous deaths annually. Since military personnel are exposed to heat, the purpose of the study was to evaluate military personnel's knowledge, attitudes, and practices (KAP) related to heat-related illnesses. Their KAP may help to prevent heat-related illness.

Methods: We conducted a cross-sectional study using a structured online questionnaire on 168 military personnel who were training and working in a high-temperature and high-humidity environment all year round in Jeddah, Saudi Arabia. The questionnaire assessed the KAP and associated factors and was distributed as a Google Form.

Results: The mean knowledge score was 9.04 (range = 2-13, SD = 1.832), the mean awareness score was 9.61 (range = 4-15, SD = 2.415), and the mean practice score was 3.39 (range = 0-6, SD = 1.703). Most participants correctly identified symptoms (n=130; 77.4%). In terms of attitudes, most participants showed a good attitude (n=151; 81%), though 24.4% did not perceive the risk. Regarding practice, most were attentive to heat-related illness signs and hydration(75.6%), but there were gaps in receiving briefings from doctors (69%) and adequate guidance on treatment (56%). There was a split opinion on whether commanders adjust field activities based on temperature warnings (54.8% Yes, 45.2% No). There were no significant differences in knowledge scores based on age or educational level (both p>0.05), while some age and education-related differences were noted in practice scores (p<0.05). There was a positive correlation between knowledge and attitudes (r = 0.222, p = 0.004), knowledge and practices (r = 0.165, p = 0.033), and attitudes and practice (r=0.326, p < 0.001).

Conclusion: Our study found that military personnel generally possess good knowledge of heat-related illnesses and good attitudes and practices concerning heat-related illnesses. However, there are areas in need of improvement, and enhancing awareness and practical implementation of preventive measures, along with the development of precise guidance and protocols, should involve active collaboration between military commanders and healthcare professionals.

## Introduction

Heat-related illnesses significantly threaten millions of people worldwide, ranging from minor forms like heat cramps to life-threatening heat stroke [1]. Heat exhaustion, a milder form, typically occurs with a body temperature between 38°C and 40°C and can result from factors like excessive exercise, exposure to high environmental temperatures, dehydration, and failure to adapt to the surroundings [2,3]. Symptoms of heat exhaustion include weakness, irritability, dizziness, vomiting, nausea, headache, diarrhea, goosebumps, and loss of coordination [4]. Immediate management involves hydration, seeking a cooler environment, rest, and monitoring for resolution, as untreated heat exhaustion can progress to heatstroke [4,5]. Heatstroke, a more severe condition, is defined by a body temperature of 40°C or higher, accompanied by central nervous system dysfunction [2,6]. It manifests with confusion, dizziness, hallucinations, delirium, seizures, tachycardia, hypotension, and multi-organ dysfunction, which can be fatal [6]. Heatstroke is differentiated from heat exhaustion by hyperthermia, conspicuous central nervous system dysfunction, and anhidrosis (lack of sweating), indicating thermoregulatory failure [7,8]. It can lead to complications like acute respiratory distress syndrome, rhabdomyolysis, intestinal ischemia, encephalopathy, electrolyte imbalances, and acute renal failure [2,9].

With global warming, the estimated global annual heat-related deaths are projected to reach 90,000 in 2030 and over 255,000 in 2050, emphasizing the need for greater awareness of these illnesses [10]. Environmental risk factors include high temperatures, humidity, and sun exposure, while individual risk factors are insufficient fluid intake, physical exertion, physical condition, medications, and pregnancy [11]. Military personnel, often exposed to high physical exertion in hot and humid conditions, face both environmental and individual risk factors for heat-related illnesses [12]. These conditions can impair their judgment, physical performance, and combat effectiveness [12,13]. Therefore, reducing heat-related illnesses is a key factor in ensuring the combat effectiveness of the military during heat waves [11].

Studies have shown that knowledge and practices among military personnel regarding heat-related illnesses are generally better compared to the general population, thanks to comprehensive training programs and preventive measures [11]. However, there are areas in which improvements can be made. Enhancing the practical application of knowledge and improving adherence to preventive measures, especially in operational settings, is critical [11]. As climate change increases the frequency and severity of extreme heat events, the military must continually adapt and refine its practices to protect the health and readiness of its personnel in challenging environments [11,14]. Studies conducted in the United States and China showed that military personnel had an overall high level of KAP of heat-related illnesses, influenced by age, military rank, educational level, and climate of their residential areas [11,15].

The only available studies were conducted on non-military participants in Saudi Arabia, involving pilgrims and the general population. They showed that pilgrims had good knowledge of heat-related illness, while a third of the general population in Jeddah had poor knowledge [16,17]. However, there is a lack of comprehensive research on heat-related illnesses among Saudi military personnel. To address this gap, this study aimed to assess the knowledge, attitudes, and practices (KAP) of military personnel regarding heat-related illness among military personnel in Jeddah, Saudi Arabia. This study is essential for understanding and potentially improving the KAP of military personnel regarding heat-related illnesses and can provide valuable insights to enhance their preparedness.

## Materials and methods

Study design

This was a cross-sectional study conducted in Jeddah, Saudi Arabia, on Military Personnel who were training and working in a high-temperature and high-humidity environment all year round at King Abdullah Air Base, King Faisal Naval Base, and King Fahad Armed Forces Hospital during the summer of 2022. The average annual temperature is 35℃ 95℉ in Jeddah. The warmest month of the year is June, with a maximum temperature of 39℃ 102℉. Regarding humidity, on average, September is the most humid month, at 67.0%. July is the least humid month, at 53.0%. The average annual percentage of humidity is 60%.

Sampling

The sample size was calculated by using the Raosoft software: http://www.raosoft.com/samplesize.html. The required sample size was estimated at the 95 percent confidence level with an estimated 50% prevalence, with a margin of error of + 5%. The required minimum sample size was determined to be 278, and we used a convenience sampling technique to recruit participants. This is a non-probability sampling technique, and it was chosen because it is the best for geographical proximity, availability of participants at a given time, or their willingness to participate.

Data collection

We used a structured questionnaire previously used in a study by Wang et al. [11]. The questionnaires were distributed to the participants as a Google Form with an invitation letter describing the study aim and a consent form. The participants signed the consent form before completing the questionnaires.

Data analysis

Collected data were entered and analyzed using the Statistical Package for Social Science (SPSS), Version 24 (IBM Corp., Armonk, NY, USA). Quantitative variables were presented in means and standard deviations or medians and quartiles depending on distribution. Categorical variables were presented in numbers and percentages and compared using the Chi-square test, as indicated. A comparison of these variables was made using an independent t-test/ANOVA, and a statistical significance cut-off of p < 0.05 was considered significant. The p-values were calculated for two-tailed tests.

Ethical considerations

The study was approved by the Research and Ethics Committee of King Fahad Armed Forced Hospital in Jeddah, Kingdom of Saudi Arabia. The military personnel's privacy and confidentiality were assured, no identifiers were collected, and all data obtained were anonymous. The data were kept private, with authorized access only to research investigators. The authors signed informed consent before completing the data, and they had the right to withdraw at any time. No incentives were provided for participation.

## Results

Participant demographics

This study received 168 responses. Table 1 shows details for demographic characteristics. Most participants were aged 38-47 (n=95; 56.5%) and held master's degrees (n=56; 33.3%).

**Table 1:** Demographic characteristics of the participants (n = 168) The data have been represented at N (%)

Characteristics	Category	N (%)
Age (years)	17-28	3 (1.8)
	28-37	29 (17.3)
	38-47	95 (56.5)
	48-57	41 (24.4)
Educational level	High school graduate	41 (24.4)
	diploma	20 (11.9)
	Bachelor's degree	51 (30.4)
	Master's degree	56 (33.3)

Response to questions on knowledge

Table 2 presents responses on knowledge of heat-related illnesses. Most participants correctly identified that fainting can occur due to heat-related illnesses during field training exercises (n=150; 89.3%). Most participants (n=150; 89.3%) recognized heat-related fainting during field exercises and cold environments, fluids, and ventilation as the appropriate first. Participants also recognized common symptoms like fever, fatigue, and chest pain and understood dehydration and lack of sweating as indicators (n=130; 77.4%). Some misconceptions, like thinking thick clothing prevents heat-related illnesses, were noted. Nonetheless, most correctly identified staying hydrated and moving victims to a cold environment as vital measures. Participants also showed awareness of risk factors, such as weight gain and alcohol consumption. While recognizing fainting as a severe symptom was encouraging, some areas, like preferring oral rehydration solution over water and identifying the most dangerous thermal disease, require further education. 

**Table 2:** Responses to knowledge items (n = 168) The data have been represented at N (%).

	Question	Category	N (%)
Yes or No responses	Can fainting due to heat-related illnesses occur during field training exercises?"	No	18 (10.7)
		Yes	150 (89.3)
	Is heat exhaustion/heat stroke treated by transporting the victim to a cold environment, drinking fluids, ice packs and ventilation?	No	18 (10.7)
		Yes	150 (89.3)
	Are fever, fatigue and chest pain common symptoms of heat exhaustion/heat stroke?	No	38 (22.6)
		Yes	130 (77.4)
	Can wearing thick clothing prevent heat exhaustion/heat stroke?"	No	138 (82.1)
		Yes	30 (17.9)
	When heat stroke is suspected, should you transfer the victim to a cold environment and then call an ambulance?	No	13 (7.7)
		Yes	155 (92.3)
	Can muscle contraction due to heat-related illnesses occur during field training exercises?	No	40 (23.8)
		Yes	128 (76.2)
	Can body cooling prevent heat exhaustion/heat stroke?	No	22 (13.1)
		Yes	146 (86.9)
	Is it possible to protect against heat stroke by staying in cold regions?	No	17 (10.1)
		Yes	151 (89.9)
	Is dehydration a symptom of heat exhaustion/heat stroke?	No	25 (14.9)
		Yes	143 (85.1)
	Does sweating lower body temperature?	No	44 (26.2)
		Yes	124 (73.8)
	Are only physically weak people susceptible to heat-related illnesses during field training exercises?	No	125 (74.4)
		Yes	43 (25.6)
	Can heat-related illnesses lead to a rapid loss of life during field training exercises?	No	46 (27.4)
		Yes	122 (72.6)
	Is heat exhaustion/heat stroke known as a body temperature above 40 degrees?	No	60 (35.2)
		Yes	108 (63.8)
Multiple-choice responses	Please identify the symptoms of heat-related illnesses that you consider severe during field training exercises	Fainting	106 (63.1)
		pooping	32 (19.0%)
		Sweating	19 (11.3)
		Lack of sweating	11 (6.5)
	Which drink do you prefer to give a person with heat exhaustion/heat stroke?	Water	103 (61.3)
		Oral dehydration treatment solution	63 (37.5)
		Soft drink	2 (1.2)
	Which of the following factors increases the risk of heat-related illnesses during field training exercises?	Aging	17 (10.1)
		Weight gain	98 (58.3)
		Drinking alcohol	37 (22.0)
		Drink enough fluids	16 (9.5)
	How can I be protected against heat-related illnesses during field training exercises?	Wear thick and dark clothing	5 (3.0)
		Use sunscreen	12 (7.1)
		Drink enough fluids	151 (89.9)
	What kind of thermal disease is the most dangerous?	Heat exhaustion	9 (5.4)
		Thermal fainting	43 (25.6)
		Heat cramping	72 (42.9)
		Heat stroke	44 (26.2)

Responses to questions on attitudes and practices

Table 3 shows the results of participants' attitudes and practices related to heat-related illnesses. A majority showed a high level of concern (n=136; 81.0%) and reported taking proactive steps in response to high-temperature warnings to prevent heat-related issues. However, 41 (24.4%) did not perceive the risk, indicating the need for targeted awareness efforts in this group of personnel. Most participants (n=109; 64.9%) believed that doctors were not doing enough to raise awareness about heat-related illnesses.

**Table 3:** Responses to attitude and practice items (n = 168) The data have been represented at N (%).

	Question	Category	N (%)
Attitude	Will you take preventive measures against heat cramping, heat exhaustion and heat stroke before and during field training if a high temperature warning is issued?	Not at all	5 (3.0)
		Sometimes	27 (16.1)
		Much	136 (81.0)
	How concerned are you about the risk of heat illness during field training?	Not at all	41 (24.4)
		I don’t know	19 (11.3)
		Little concern	75 (44.6)
		Very concerned	32 (19.0)
	Do you consider yourself sensitive to extreme heat?	Not at all	33 (19.6)
		I don’t know	27 (16.1)
		Somewhat	85 (50.6)
		Very much	23 (13.7)
	Do you think doctors are raising sufficient awareness of the risk of heat disease?	Too little	109 (64.9)
		I don’t know	20 (11.9)
		Just enough	24 (14.3)
		Too much	15 (8.9)
Practice	Do your commanders manage field activities at a relatively cooler time when a temperature warning is issued?	No	76 (45.2)
		Yes	92 (54.8)
	Before going out for field exercises, do doctors tell you ways to protect against heat-related illnesses and how to deal with them?	No	116 (69.0)
		Yes	52 (31.0)
	When you go out for field training, do doctors prepare good measures to treat heat-related illnesses?	No	94 (56.0)
		Yes	74 (44.0)
	Do you pay attention to the signs and symptoms of heat cramps, heat exhaustion and heat stroke?	No	41 (24.4)
		Yes	127 (75.6)
	Do you only drink water when you feel thirsty during field training?	No	47 (28.0)
		Yes	121 (72.0)
	When your unit goes out for field training exercises, do doctors prepare good measures to treat heat-related illnesses, such as medications, fluids, and temperature lowering devices?	No	64 (38.1)
		Yes	104 (61.9)

Most participants paid attention to heat-related illness signs (n=127; 75.6%) and avoided waiting until thirsty to drink water (n=121; 72%). However, there are areas for improvement: 116 (69%) did not receive briefings from doctors on protection against heat-related illnesses before field exercises, and 94 (56%) lacked adequate guidance on treatment. There was a split opinion on whether commanders adjust field activities based on temperature warnings.

Mean scores for KAP

**Figure 1:**
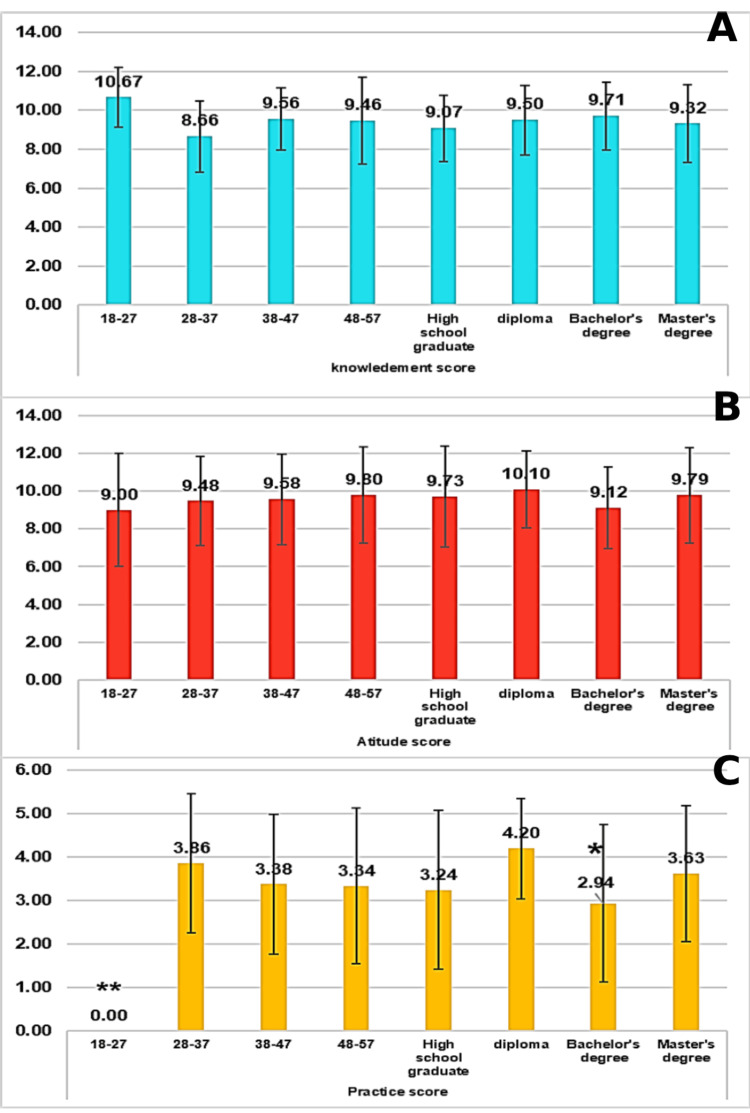
Mean KAP scores (A-C) according to demographic characteristics The data have been represented as mean ± SD. P-values were calculated by a one-way ANOVA. P-value is considered significant (*p < 0.05, **p < 0.01).

The detailed mean KAP scores (K-, A-, and P-scores, respectively), as well as the mean overall scores according to different demographic characteristics, are illustrated in Figure 1. The mean K-score was 9.04 (range = 2-13, SD = 1.832). There were no significant differences in mean K-score according to age and educational level (p > 0.05). In addition, the mean A-score was 9.61 (range = 4-15, SD = 2.415). There were no significant differences in mean K-score according to age and educational level (p > 0.05).

On the other hand, the mean P-score was 3.39 (range = 0-6, SD = 1.703). Participants aged 18-27 exhibited significantly lower P-scores (Mean= 0.001, SD= 0.001) compared to those in the 28-37, 38-47, and 48-57 age groups (p < 0.01. Additionally, participants with a bachelor's degree had significantly lower P-scores (Mean= 2.94, SD= 1.81) than those with a diploma (p < 0.05).

Correlations between KAP

Correlation analyses suggested a significant positive correlation between K- and A-scores (r = 0.222, p = 0.004), K- and P-scores (r = 0.165, p = 0.033), and A- and P-scores (r = 0.326, p < 0.001) (Table 4).

**Table 4:** Correlations between knowledge, attitude, and practice scores The p-value is calculated by a Pearson correlation test. P-value is considered significant (*p < 0.05, **p < 0.01).

		Knowledge score	Attitude score	Practice score
Knowledge score	R	1		
	P-value			
Attitude score	R	0.222^**^	1	
	P-value	0.004		
Practice score	R	0.165^*^	0.326^**^	1
	P-value	0.033	<0.001	

## Discussion

Heat-related illnesses are a significant concern for military personnel, especially those serving in hot and arid climates in the Middle East region and Saudi Arabia in particular. Understanding the KAP of military personnel regarding heat-related illnesses is crucial for ensuring their health and operational readiness in challenging environments. This study assessed the KAP towards heat-related illnesses’ symptoms, treatment, and prevention among the military personnel in Jeddah City, Saudi Arabia.

Our findings showed that most participants (89%) had a good knowledge of symptoms, treatment, and prevention measures of heat-related illnesses. This is higher compared to almost 70% reported among the general public in Jeddah [17]. Good knowledge might be attributed to military training that encompasses a wide area of topics, especially emergencies and how to give first aid services, which extensively cover heat-related illnesses. Military personnel typically receive training and education on heat-related illnesses as part of their basic training and ongoing professional development, which might explain their higher knowledge [18]. However, we found some misconceptions about heat-related illnesses, like thinking thick clothing prevents heat-related illnesses. Similarly, other previous studies reported knowledge gaps. El Gamal et al. found that 46.4% of pilgrims believed thirstiness was the only sign, and 34% did not know that sunscreen is protective [17]. We found that some personnel did not know that staying hydrated and moving victims to a cold environment were vital prevention and management measures. Other studies indicated that in some cases, personnel may be unaware of the importance of maintaining proper hydration and the significance of acclimatization, which makes them more susceptible to heat stress [19]. On the other hand, military personnel may be aware of the need for regular fluid intake but may not consistently adhere to this guidance during exercises or operations. This inconsistency can put them at greater risk for heat-related illnesses [20]. Our findings highlight the need to reinforce accurate information for improved awareness and preparedness during heat-related emergencies.

Though most participants showed good attitudes and practices regarding proactive prevention, almost a quarter of participants did not perceive the risk, and did not take measures, similar to the findings of a previous study among Hajj pilgrims [15]. Our findings align with a previous study conducted on military personnel in China, indicating that most reported the necessity for maintaining good preventive measures [11]. Almost two-thirds of participants believed that doctors were not raising enough awareness about heat-related illnesses. This might be explained by poor awareness among doctors, as a previous study conducted in Saudi Arabia suggested. Aljumaan et al. [21] found that when heat stroke was listed alongside other heat-related disorders, nearly two-thirds of medical workers could correctly identify its definition. However, heat stroke definition knowledge, on the other hand, was much lower among both health and non-health personnel. This indicates the need for encouraging training healthcare providers about heat-related illness and their involvement in raising awareness. 

Three-quarters of the participants in our study had good practice, similar to military personnel in China [11]. On the training field, most reported the lack of measures and guidance on protection against heat-related illnesses and treatment. Those who thought that commanders adjust field activities based on temperature warnings were equal to the opponents (54.8% vs 45.2%), highlighting the need for more consistent protocols and practices within the military units. In contrast, a Chinese study reported that most (64.8%) participants were educated prior to field training [11]. We found that 28% of participants did not drink water when they felt thirsty on the training field despite awareness of the benefits. This might indicate the difference between knowledge and implementation. It was found that most pilgrims had appropriate practice at home, while they had wrong practice while outdoors [17]. Similarly, it was found that military personnel are more likely to follow preventive measures during training exercises compared to combat operations [22]. This suggests a need for more realistic training scenarios and enhanced integration of heat stress management into operational planning. We found no There were no significant differences in knowledge and awareness according to age and educational level (both P > 0.05). In contrast, among pilgrims in Saudi Arabia, advanced age exhibited a strong and statistically significant positive association with an increased KAP score (b=0.177, p<0.001), and the mean KAP score was significantly higher among females when compared to males (b=-2.25, p <0.001). Jeddah general public with health education related to heat-related illnesses exhibited a significantly higher KAP score compared to those who had not received such education (b=2.327, p< 0.001) [17]. Our findings also disagree with a Chinese study where there were significant differences in mean knowledge score according to age and educational level (p < 0.05) [11]. A study on Hajj pilgrims showed that the 48-57 years age group was two times more likely to have good knowledge than the <38 years age group (adjusted odd ratio (aOR): 2.02, 95%CI = 1.45-2.83, p<0.001).

Our findings align with other previous studies [11,16,17] by showing significant differences in practice scores according to age and education. Participants aged 18-27 exhibited significantly lower practice scores compared to those in the 28-37, 38-47, and 48-57 age groups (p < 0.01). This contrasts the findings among pilgrims, showing that those aged 38 years and above had the lowest practice scores [16]. There is evidence that young military recruits (aged 16 to 19 years), exhibit a higher vulnerability to heat illnesses compared to older adults, suggesting the need for enhanced risk reduction measures in this demographic group. It was suggested that this high prevalence in this group might be attributed to physical demands, military status, education, and selection procedures rather than disparities in the physiological characteristics between adults and adolescents [18]. We found a significant positive correlation between knowledge and awareness scores (r = 0.222, p = 0.004 knowledge and practice scores (r = 0.165, p = 0.033), and awareness and practice scores (r = 0.326, p < 0.001), which aligns with studies conducted Saudi Arabia [16], and in China [11,23]. These findings emphasize the necessity of targeted educational and awareness initiatives, consistent communication from healthcare professionals, and the establishment of clear protocols to ensure the safety and well-being of military personnel during field training exercises and real-life operations, especially in the context of rising concerns about heat-related illnesses. Addressing these areas could significantly enhance military preparedness and reduce the risks associated with heat-related emergencies.

This study had limitations to consider. Our sample size analyzed was small as other eligible participants did not respond, which constitutes a minor fraction of the entire military population in Jeddah and Saudi Arabia in general. The study's cross-sectional design restricts the identification of the exposure-effect relationship. Furthermore, since we gathered data through an online questionnaire, the responses obtained are susceptible to potential information bias. The questionnaire utilized in our study has not undergone formal validation in our study though it was previously validated by Wang et al. [11]. While our findings explore the participants' knowledge of heat-related illnesses, we recognize that the lack of a validated questionnaire in the context of our study may impact the overall robustness of our data. Additionally, as we did not directly observe the practices, the results may not provide an entirely accurate reflection of the actual behaviors and practices reported by participants. We also relied on self-reported data, prone to under- and over-reporting. The study was limited to Jeddah and may not reflect the diverse conditions across different regions or the practices of military personnel in varied climates. To mitigate these limitations, we recommend further longitudinal studies extending to more military institutions to have larger samples.

## Conclusions

Our study showed overall good knowledge, attitude, and practices of military personnel regarding heat-related illness. However, there are areas of weakness that require improvements, such as tackling some misconceptions, targeted awareness and education efforts, the establishment of clear protocols, adjusting field activities based on temperature warnings, and engagement of military commanders and healthcare providers to increase KAP among military personnel regarding symptom detection, treatment and prevention of heat-related illnesses, especially on the fields or during operations. Therefore, enhancing the dynamically practical application of knowledge and improving adherence to preventive measures, especially in operational settings, with a continuous adaption to global warming, would improve the health and readiness of military personnel.
